# The application of varying amount of green manure combined with nitrogen fertilizer altered the soil bacterial community and rice yield in karst paddy areas

**DOI:** 10.1186/s12870-024-05351-7

**Published:** 2024-07-08

**Authors:** Juxin Zhong, Zhongyi Li, Hongqin Tang, Wenbin Dong, Caihui Wei, Tieguang He

**Affiliations:** 1 Key Laboratory of Karst Dynamics, Ministry of Natural Resources & Guangxi, Institute of Karst Geology, Key Laboratory of Karst Ecosystem and Treatment of Rocky Desertification, Ministry of Natural Resources, Chinese Academy of Geological Sciences, Guilin, Guangxi 541004 China; 2 Agricultural Resource and Environment Research Institute, Guangxi Academy of Agricultural Sciences/Guangxi Key Laboratory of Arable Land Conservation, Nanning, Guangxi 530007 China

**Keywords:** Green manure, Bacterial community, Karst, Co-occurrence network, Rice yield

## Abstract

Long-term application of green manure (GM) and nitrogen (N) fertilizers markedly improved soil fertility and boosted rice yield in ecologically fragile karst paddy fields. However, the precise response mechanisms of the soil bacterial community to varying amounts of green manure alone and in combination with N fertilizer in such environments remain poorly elucidated. In this study, we investigated the soil bacterial communities, keystone taxa, and their relationship with soil environmental variables across eight fertilization treatments. These treatments included group without N addition (N_0_M_0_, no N fertilizer and no GM; N_0_M_22.5_, 22.5 t/ha GM; N_0_M_45_, 45 t/ha GM, N_0_M_67.5_, 67.5 t/ha GM) and group with N addition (NM_0_, N fertilizer and no GM; NM_22.5_, N fertilizer and 22.5 t/ha GM; NM_45_, N fertilizer and 45 t/ha GM; NM_67.5_, N fertilizer and 67.5 t/ha GM). The results revealed that increasing green manure input significantly boosted rice yield by 15.51–22.08% and 21.84–35% in both the group without and with N addition, respectively, compared to N_0_M_0_ treatment. Moreover, with escalating green manure input, soil TN, AN, AK, and AP showed an increasing trend in the group without N addition. However, following the addition of N fertilizer, TN and AN content initially rose, followed by a decline due to the enhanced nutrient availability for rice. Furthermore, the application of a large amount of N fertilizer decreased the C: N ratio in the soil, resulting in significant changes in both the soil microbial community and its function. Particularly noteworthy was the transition of keystone taxa from their original roles as N-fixing and carbon-degrading groups (oligotrophs) to roles in carbon degradation (copiotrophs), nitrification, and denitrification. This shift in soil community and function might serve as a primary factor contributing to enhanced nutrient utilization efficiency in rice, thus significantly promoting rice yield.

**Supplementary Information:**

The online version contains supplementary material available at 10.1186/s12870-024-05351-7.

## Introduction

The global advocacy for green and clean energy aims to mitigate the environmental toxicity caused by chemical fertilizers [1]. Chinese Milk vetch (*Astragalus sinicus* L.) is frequently utilized as a leguminous green manure in rotation with rice in southern China, markedly diminishing environmental risks while enhancing soil fertility and rice yields [2]. Studies have demonstrated that incorporating green manure can effectively substitute 20–40% of the chemical N fertilizers, presenting a highly efficient approach for optimizing fertilizer application [3].

Green manure is typically sown during the winter fallow season and subsequently integrated into the paddy field at their blooming period [4]. The application of green manure significantly enhanced nutrient availability and hence improved rice yield [5]. This improvement can be attributed to the nutrient-rich composition of green manure, including N, P and K, as well as gradual release of atmospheric N fixed in its roots during the decay process, ensuring a steady supply of nutrients for subsequent growth of rice [6]. However, research has emphasized significant differences in rice utilization efficiency among different amounts of green manure and fertilizer inputs [7], yet the underlying reason for such variations remain unclear. One plausible explanation is that organic materials released nutrients relatively slowly, whereas early-stage fertilization can rapidly supply nutrients to rice [8]. Over time, as green manure decomposes and releases nutrients, rice can continuously absorb them, thereby enhancing its balanced nutrient supply capability, particularly the stable N supply in the soil [9]. Further investigation revealed that the degradation of green manure and the conversion of N by soil microorganisms may be more critical factors influencing rice nutrient absorption [10].

Soil microorganisms played critical roles in decomposing green manure, thereby releasing nutrients that can be absorbed and utilized by rice [11, 12]. Studies have shown that applying green manure promoted microbial growth and reproduction, thereby facilitating the release of nutrients from green manure [13, 14].Taxa such as Proteobacteria, Bacteroidetes, and Ascomycota, which thrive in nutrient-rich environments, were particularly stimulated by this process, thus improving rice’s efficiency in utilizing green manure [15–17]. Green manure is commonly used in combination with fertilizers. However, the addition of exogenous N significantly reduced the soil C/N ratio and disturbed soil nutrient patterns [18], affecting microorganisms’ access to available resources and altering the overall composition [19, 20] and function of keystone taxa within the microbial community [15, 21], ultimately impacting rice yield. Keystone taxa play a crucial role in regulating microbial community structure and function, and co-occurrence network can help us identify them [22, 23]. The decrease in complexity of the network may potentially result in the loss of microbial function of keystone taxa. Many pieces of evidence have shown that excessive loading of N critically reduced the diversity of microbial community, inhibiting N fixation [24], as well as the nitrification and denitrification capacity of certain bacterial functional groups [25, 26]. Therefore, comprehending the impact of different amounts of green manure, especially when combined with N fertilizer, on microorganisms and their functions is crucial for understanding their roles in enhancing the utilization efficiency and yield of rice.

Carbonate rocks are widely distributed in karst areas [27], hosting bacterial communities on their surfaces that play pivotal ecological roles, including N fixation, nitrate metabolism, and carbon-inorganic compound metabolism [28]. Calcareous soil derived from carbonate dissolution and weathering exhibits alkaline and calcium-rich characteristics [29], harboring unique microbial communities with distinct functionalities [30]. The application of green manure and chemical fertilizers can reduce soil pH, thereby influencing the soil environment and subsequently impacting the microbial community [31, 32]. This alteration may lead to the proliferation of specific functional microorganisms, consequently affecting soil element cycling and nutrient uptake by rice plants, ultimately influencing rice yield. Based on the aforementioned findings, we formulated a scientific hypothesis positing that the cultivation of green manure alone or in combination with N fertilizer in karst paddy fields would promote rice yield by modifying the soil nutrient and affecting the composition and function of the soil microbial community.

Herein, we present the methodology and results of a three-year-long field experiment conducted to assess the effects of different fertilization regimes on rice yields, soil nutrients, and the soil bacterial community in typical brownish-yellow soil within karst regions. Our primary objectives were to address the following four questions: (1) What are the effects of varying amounts of green manure, both independently and in combination with N fertilization, on soil nutrient and rice yield? (2) How do different fertilization regimes affect bacterial community diversity and structure, as well as keystone taxa? (3) What are the relationships between soil nutrients, the soil bacterial community, and rice yield? (4) How does the interaction between fertilization regimes and microbial dynamics influence rice productivity? Through rigorous experimentation and analysis, we aimed to provide comprehensive insights into the complex interplay between fertilization regimes, soil microbial dynamics, and rice productivity in karst paddy environments.

## Materials and methods

### Site description

The experimental site is located in Nanning County, Guangxi Province, China (107° 51’21’’ E, 23°0’ 41’’ N). The region has a subtropical monsoon climate with an annual average temperature of 21.6℃, precipitation of approximately 1,300 mm, and an average altitude of 64 m. The soil was classified as brown-yellow lime soil derived from carbonate rock salt. The basic physical and chemical properties of the soil at a depth 0–20 cm are as follows: pH 7.03, soil organic carbon (SOC) 17.4 g/kg, total nitrogen (TN) 1.96 g/kg, available nitrogen (AN) 158.1 mg/kg, available phosphorus (AP) 11.7 mg/kg, and available potassium (AK) 86 mg/kg. The N content of green manure was 32.3 g/kg.

### Experimental design and plant material

The experiment was initiated in 2017, implementing a double-cropping system for rice cultivation. The rice variety used, Guiyu 9, was obtained from the Rice Research Institute of the Guangxi Academy of Agricultural Sciences. The green manure variety, Chinese milk vetch (*Astragalus sinicus* L.), with the seed name Guizi 7, was sourced from the Agricultural Resources and Environment Research Institute of the Guangxi Academy of Agricultural Sciences. The milk vetch was uniformly sown 1–2 weeks before the late rice harvest, cultivated during the winter fallow season, and subsequently incorporated into the paddy field at the peak of its blooming [4]. Due to insufficient in-situ green manure, milk vetch was harvested and weighed from an alternative site, achieving return amounts of 45 t/ha and 67.5 t/ha of green manure to the field, respectively.

A total of eight treatments were administered: the group without N addition, which included (i) no N fertilizer and no GM (N_0_M_0_), (ii) 22.5 t/ha GM (N_0_M_22.5_), (iii) 45 t/ha GM (N_0_M_45_), and (iv) 67.5 t/ha GM (N_0_M_67.5_). The group with N addition included (v) N fertilizer and no GM (NM_0_), (vi) N fertilizer and 22.5 t/ha GM (NM_22.5_), (vii) N fertilizer and 45 t/ha GM (NM_45_), and (viii) N fertilizer and 67.5 t/ha GM (NM_67.5_). We employed a randomized-block design, with three replications of each treatment. Each experimental plot area was 16.5 m^2^ (3.3 m × 5 m), separated by ridges to prevent water and nutrient movement between plots. The fertilizers used were urea (containing 46.4% Nitrogen), calcium superphosphate (containing 18.0% P_2_O5), and potassium chloride (containing 60% K_2_O), respectively. The N fertilizer applied to the rice was 195 kg/ha in the first year and 180 kg/ha in the second and third years. The phosphorus (P) and potassium (K) application rates remained consistent each year, with 90 kg/ha of phosphorus and 120 kg/ha of potassium. Forty percent of the N, P, and K fertilizers were applied as a basal application, while the remaining 60% were divided equally for top-dressing at the tillering and jointing-booting stages.

#### Soil sampling and physicochemical analysis

Soil samples were collected from the surface soil (0–20 cm) of each plot in July 2020. To reduce variability, five soil cores were collected from each plot using the “S” method and mixed together to form one sample. A total of 24 soil samples were transported back to the laboratory, where rice roots and stones were removed. The sampled soil was divided into two parts: one part was stored at – 80 °C for microbial sequencing, and the other part was air-dried for soil physicochemical analysis.

Soil pH was tested using the potentiometric method with a soil-to-water ratio of 1:2.5 (weight: volume). Soil organic carbon (SOC) was measured using the potassium dichromate and sulfuric oxidation method. Total nitrogen (TN) was tested using the automatic Kjeldahl method. Total phosphorus (TP) was tested using the molybdenum-antimony colorimetric method. Available N (AN) was determined using the ferrous sulfate-reducing agent diffusion method. Available phosphorus (AP) was measured using the molybdenum-antimony counterstain method with sodium bicarbonate extraction. Available potassium (AK) was measured using ammonium acetate exchange flame photometry. Exchangeable calcium (E-Ca) and exchangeable magnesium (E-Mg) were determined by ammonium acetate exchange-atomic absorption spectrophotometer. Soil physicochemical analyses were conducted according to the methods described by Lu [33].

### DNA extraction and bioinformatic analysis

Soil DNA was extracted from 2.5 g of flesh soil using the PowerSoil DNA Isolation Kit for Soil (Mobio Laboratories, Inc., Carlsbad, CA, USA). The V4–V5 fragment of the bacterial 16S rRNA genes was amplified with the primer pair 515F (5’-GTGCCAGCMGCCGCGGTAA-3’) and 907R (5’-CCGTCAATTCMTTTRAGTTT-3’) [34]. The PCR amplification conditions were denaturation at 95 °C for 10 s, annealing at 55 °C for 30 s, and extension at 72 °C for 45 s. The 16S rRNA sequences were then conducted on the Illumina NovaSeq high-throughput sequencing platform by MAGIGENE (Guangdong, China).

Forward and reverse sequences were spliced using the FLASH 1.2.11 software, and low-quality sequences (average quality score lower than 200_bp) and chimeras were removed. The remaining high-quality reads were aligned and clustered into operational taxonomic units (OTUs) with a similarity level of 97% using USEARCH software. The representative OTUs were then compared with the SILVA 132 16 S rRNA databases to determine the taxa of each sample. To eliminate potential bias caused by different sequencing depths, the OTU tables were rarefied to the minimum read number of all samples (each with 36,263 reads after quality control). Alpha diversity indices and beta diversity distance matrices were calculated using the QIIME software [35], based on the randomly sampled OTU tables with the same sequence depth. Phyla and classes with a relative abundance of ≥ 1% were defined as dominant phyla and class [15]. The 16S rRNA gene sequences were deposited into the NCBI Sequence Read Archive database with the number PRJNA1031136.

### Statistical analysis

One-way ANOVA and two-way ANOVA were conducted using SPSS 25.0 software to compare the differences in rice yield, soil properties, and alpha diversity among different treatments. Principal Component Analysis (PCA) and Analysis of Similarity (ANOSIM) were conducted with the R package “vegan” to assess the differences in soil bacterial community compositions. Redundancy analysis (RDA) using CANOCO 5.0 and Mantel test using R package “devtools” were used to evaluate correlations between bacterial community and soil factors. Structural equation modeling (SEM) was employed to analyze the potential direct and indirect effects of soil factors and microbial factors on rice yield caused by fertilization [21, 36]. The SEM analysis was conducted using the robust maximum likelihood evaluation method in AMOS 28.0 (AMOS IBM, USA) [37].

### Network analyses and keystone species

Co-occurrence networks were used to assess microbial complexity and identify potential keystone taxa. To avoid spurious correlations, soil bacteria OTUs with a relative abundance greater than 0.1% underwent Spearman correlation analysis, corrected using false discovery rate correction [38]. The R package “ psych” was used to construct the correlation network, with correlation coefficients above 0.6 and p-values below 0.05 were regarded as elements of networks [39]. Networks were then visualized using Gephi [40]. The OTUs with the highest degree and highest closeness centrality were identified as keystone taxa [22, 23, 41], and the sum of these two values was transformed into a Z-score [42]. Z-score values greater than 1.0 were selected as keystone taxa.

## Results

### Rice yield and soil properties

**Fig. 1:**
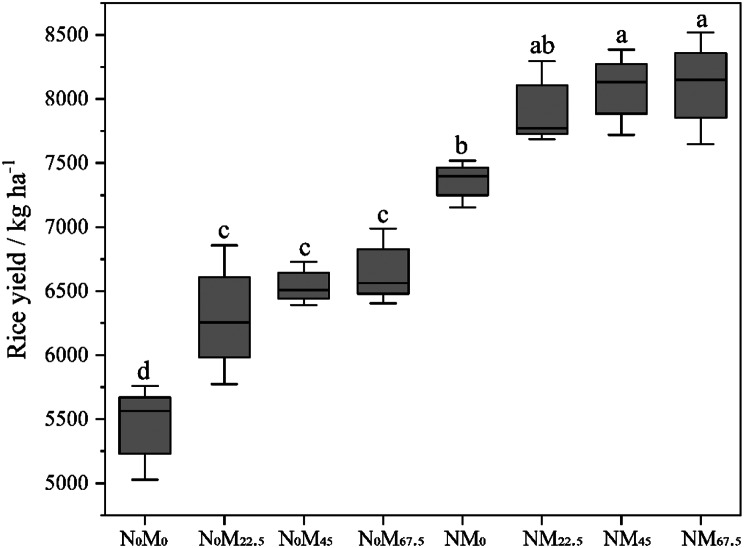
Early rice yields in different treatments (N_0_M_0_, N_0_M_22.5_, N_0_M_45_, and N_0_M_67.5_ belong to the group without N addition, and NM_0_, NM_22.5_, NM_45_, and NM_67.5_ belong to the group with N addition)

Generally, as the application of green manure increased, rice yield exhibited an upward trend (Fig. 1). Compared to N_0_M_0_ treatment, N_0_M_22.5_, N_0_M_45_, and N_0_M_67.5_ significantly increased rice yields by 15.51 to 22.08%. NM_45_ and NM_67.5_ also significantly increased the rice yields by 9.81% and 10.17% compared to NM_0_. Moreover, the addition of N significantly increased rice yield by 21.84 to 35% compared to treatments without N addition.

Two-way ANOVA revealed significant interactions between milk vetch (MV) and N fertilizer for most of the soil properties (Table 1). Across all treatments, there was a significant increase in TN and E-Mg contents compared to N_0_M_0_, alongside a notable decrease in soil pH value. The soil available nutrients (AN, AK, and AP) increased with escalating green manure application in the group without N addition. In contrast, available nutrients (AN and AP) in the group with N addition were slightly higher than those without N addition, with significant differences observed in NM_22.5_ and NM_45_ treatments. Fertilization treatments did not significantly change the SOC and TP content.

**Table 1:** Soil properties in different fertilization treatments

Treatment	pH	SOC (g/kg)	TN (g/kg)	TP (g/kg)	AN (mg/kg)	AP (mg/k)	AK (mg/kg)	E-Ca (cmol/kg)	E-Mg (cmol/kg)	C/*N*
N_0_M_0_	6.94 ± 0.02a	17.62 ± 0.34b	2.04 ± 0.04c	0.81 ± 0.02b	131.32 ± 0.66b	16.90 ± 0.96c	124.74 ± 1.88b	8.35 ± 0.15c	0.57 ± 0.02c	8.64 ± 0.12a
N_0_M_22.5_	6.76 ± 0.02b	19.16 ± 0.16ab	2.22 ± 0.02b	0.86 ± 0.02a	131.83 ± 14.73b	19.57 ± 0.75c	129.79 ± 10.01b	10.51 ± 1.80ab	0.77 ± 0.04ab	8.34 ± 0.12ab
N_0_M_45_	6.80 ± 0.03ab	18.38 ± 0.50ab	2.30 ± 0.05b	0.79 ± 0.01b	147.91 ± 4.69b	20.11 ± 1.77c	151.44 ± 11.30ab	14.09 ± 0.77a	0.77 ± 0.04ab	8.29 ± 0.12ab
N_0_M_67.5_	6.71 ± 0.05b	19.36 ± 0.84ab	2.47 ± 0.07ab	0.85 ± 0.02b	167.01 ± 5.96ab	23.78 ± 1.87c	170.48 ± 11.95a	12.56 ± 1.22ab	0.83 ± 0.04a	7.82 ± 0.12bc
NM_0_	6.49 ± 0.07c	18.73 ± 0.40ab	2.24 ± 0.06b	0.79 ± 0.00b	159.45 ± 5.8ab	46.86 ± 3.97a	121.63 ± 16.70b	11.27 ± 0.23b	0.78 ± 0.04ab	8.38 ± 0.23ab
NM_22.5_	6.45 ± 0.10c	19.17 ± 0.46ab	2.58 ± 0.04a	0.80 ± 0.01b	170.62 ± 2.97a	40.85 ± 2.75ab	103.99 ± 2.42b	11.47 ± 0.17ab	0.76 ± 0.01ab	7.44 ± 0.17c
NM_45_	6.45 ± 0.07c	18.92 ± 0.81ab	2.44 ± 0.05ab	0.818 ± 0.01ab	170.94 ± 5.61a	37.90 ± 2.32b	132.55 ± 14.99b	8.21 ± 0.40c	0.74 ± 0.01b	7.76 ± 0.27bc
NM_67.5_	6.58 ± 0.12c	19.71 ± 1.01a	2.34 ± 0.07b	0.82 ± 0.02ab	161.64 ± 8.68ab	46.53 ± 4.71a	161.20 ± 17.04ab	8.76 ± 1.07bc	0.74 ± 0.01ab	8.41 ± 0.22ab
Two-ANOVA analysis (P value)										
MV	0.219	0.188	< 0.001	0.064	0.082	0.165	0.115	0.488	0.008	0.020
N	< 0.001	0.270	0.002	0.117	0.001	< 0.001	0.004	0.040	0.281	0.044
MV × N	0.184	0.844	0.006	0.071	0.045	0.192	0.793	0.001	0.001	0.006

SOC, soil organic carbon; TN, total nitrogen; TP, total phosphorus; AN, available nitrogen; AP, available phosphorus; AK, available potassium

### Microbial community characteristics under different treatments

**Fig. 2:**
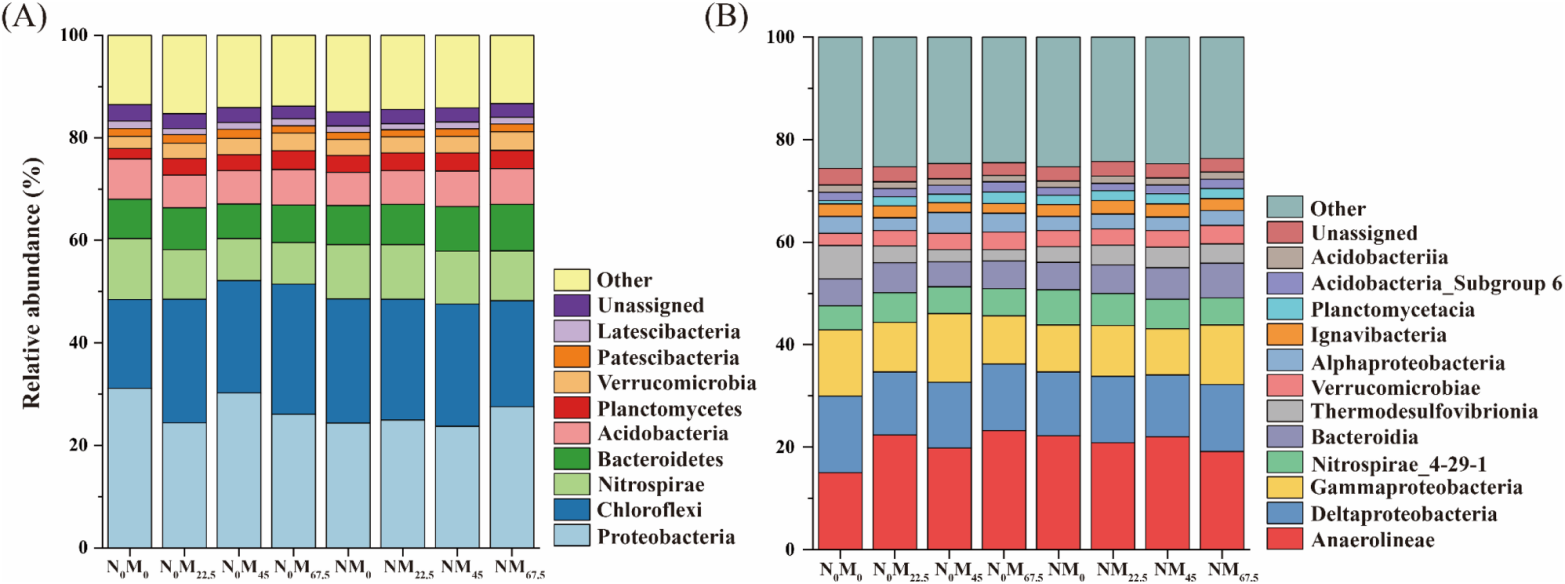
Soil bacterial community under different fertilization treatments. (A-B) The relative abundance of soil bacteria at the taxonomic levels of phylum and class, respectively

A total of 9 major phyla were identified as the dominant phyla, with the top five bacterial phyla being *Proteobacteria* (23.73–31.15%), *Chloroflexi* (17.33–25.43%), *Nitrospirae* (8.09–11.86%), *Bacteroidetes* (6.75–9.10%), and *Acidobacteria* (6.53– 7.86%; Fig. 2A). N_0_M_22.5_ and N_0_M_67.5_ significantly decreased the relative abundance of *Proteobacteria* by 21.60% and 16.33%, respectively, compared to N_0_M_0_. Similarly, N_0_M_45_ and N_0_M_67.5_ significantly decreased the relative abundance of *Nitrospirae* by 30.75% and 31.78%, respectively, compared to N_0_M_0_. Moreover, N_0_M_22.5_, N_0_M_45_, and N_0_M_67.5_ significantly decreased the relative abundance of Firmicutes by 44.76 − 77.28%, respectively, compared to N_0_M_0_. In contrast, N_0_M_22.5_, N_0_M_45_ and N_0_M_67.5_ significantly increased the relative abundance of *Chloroflexi*, *Planctomycetes* and *Verrucomicrobia* by 18.79–46.70%, 51.06–81.27%, and 26.01–45.11%, respectively, compared with N_0_M_0_. Conversely, NM_22.5_ and NM_67.5_ significantly increased the relative abundance of *Proteobacteria* by 14.84% and 16.27%, while significantly decreasing the relative abundance of *Chloroflexi* by 9.43% and 14.76% respectively, compared to NM_0_. NM_45_ and NM_67.5_ also significantly increased the relative abundance of *Bacteroidetes* by 12.00% and 13.38%, respectively, compared with NM_0_. At the class level, 12 major classes were identified as dominant class (Fig. 2B). All treatments significantly increased the relative abundance of *Anaerolineae* and *Nitrospirae_4-29-1*, compared to N_0_M_0_, while they significantly decreased the relative abundance of *Deltaproteobacteria* and *Thermodesulfovibrionia*.

### Bacterial community diversity and structure under different treatments

**Fig. 3:**
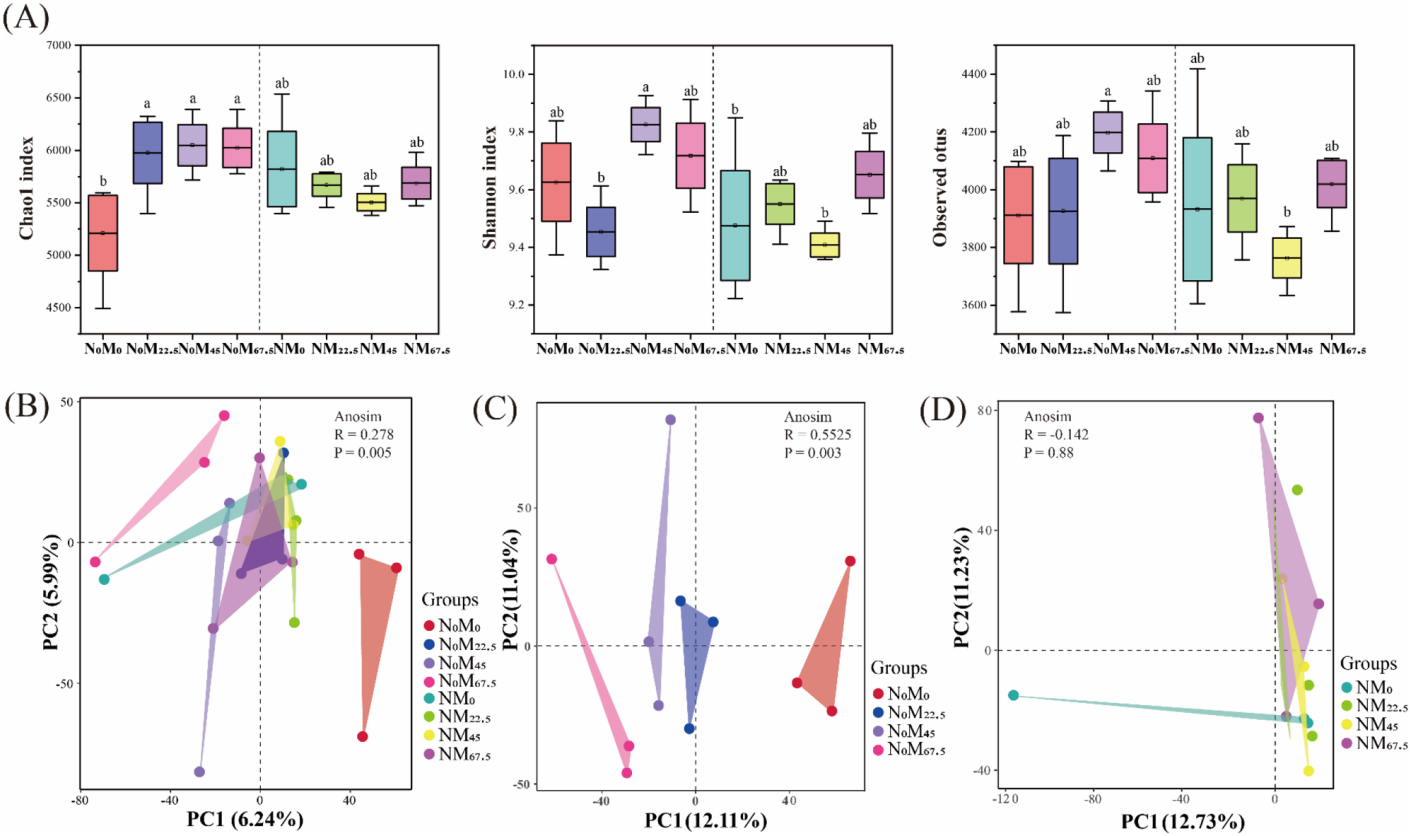
Soil bacterial diversity under different fertilization treatments. (**A**) Alpha diversity measures including the Chao1 index, Shannon index, and observed OTUs. (**B**-**D**) Principal component analysis (PCA) plot showing bacterial community structures in different treatments, respectively. Different lowercase letters indicate significant differences (*p* < 0.05). ANOSIM indicates the difference in community composition between treatments, with *R* > 0 indicating significant differences between groups, and *R* < 0 indicating greater differences within groups than between groups

The alpha and beta diversities of soil bacteria are depicted in Fig. 3. Compared to the N_0_M_0_ treatment, the Chao1 index values were significantly increased in the group without N addition (N_0_M_22.5_, N_0_M_45_ and N_0_M_67.5_), while there was no significant difference in the group with N addition (NM_22.5_, NM_45_ and NM_67.5_) (Fig. 3A). Additionally, the highest Shannon index was observed in N_0_M_45_, while the lowest values were observed in NM_45_. Similarly, the observed OTUs showed consistent results with the Shannon index.

Fertilization regimes significantly affected the soil bacterial community structure. The PCA results revealed that the soil bacterial community treated with N_0_M_0_ treatment was significantly separated from those treated with N_0_M_45_ and N_0_M_67.5_ treatments, whereas overlap was observed among N_0_M_22.5_, NM_0_, NM_22.5_, NM_45_ and NM_67.5_ treatments (Fig. 3B). Significant differences in the community structure of soil bacteria were evident in the group without N addition (Fig. 3C), while the community structure was similar in the group with N addition (Fig. 3D).

### Co-occurrence network and keystone taxa under different treatments

Network analysis was used to reveal the interactions of soil bacteria across varied fertilization treatments. As green manure application increased, species transfers between modules occurred, leading to enhanced stability in the co-occurrence network of soil bacteria, irrespective of N fertilizer application (Fig. S1). Moreover, in the absence of N addition, the proportion of negative correlation decreased with escalating green manure input, while it tended to increase in the presence of N addition (Table S1). This suggests that green manure primarily exerted a synergistic effect on soil bacterial interaction, whereas competition became the dominant effect following N fertilizer addition.

**Fig. 4:**
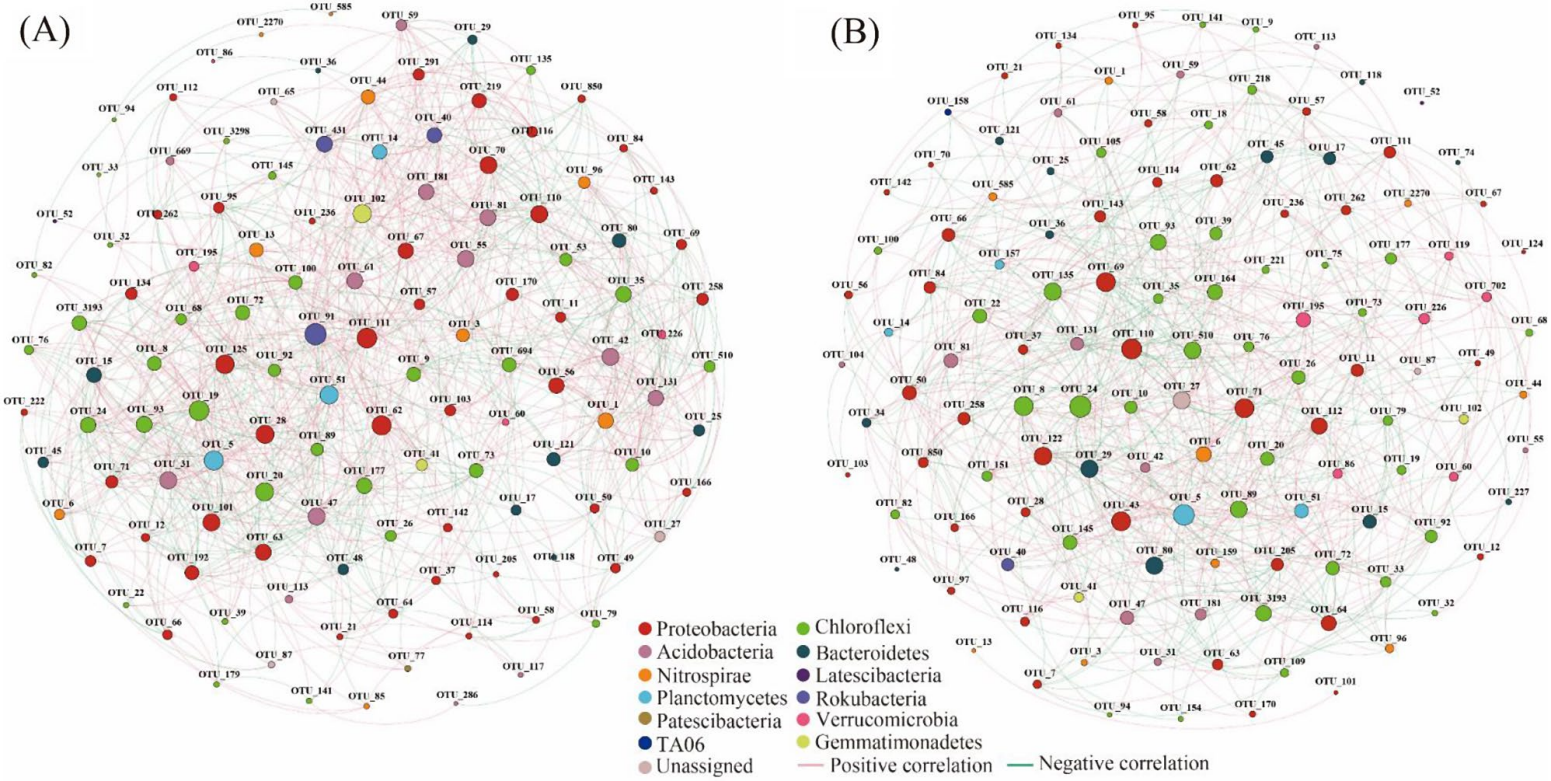
Soil bacterial co-occurrence network diagram. (**A**-**B**) Bacterial interactions of the group without N addition and the group with N addition, respectively. Positive correlations are displayed in red, while negative correlations are displayed in green. Nodes are colored according to different species categories. The size of each node represents the average abundance of the species

Eight treatments were categorized into two groups to identify keystone taxa (Fig. 4). In the group without N addition, keystone taxa included *Latescibacteria* (OTU91, 431), Anaerolineaceae (OTU19, 20, 93) from *Chloroflexi*, *Betaproteobacteriales* (OTU125, 110, 63) and *Ectothiorhodospirales* (OTU28) from *Gammaproteobacteria*, *Myxococcales* (OTU111,70), *Desulfarculales* (OTU101) from *Deltaproteobacteria*, *Rhizobiales* of *Alphaproteobacteria*, *Pla4 lineage* (OTU51) of *Planctomycetes*, *Gemmatimonadales* of *Gemmatimonadetes*, and *Subgroup* (4, 5, 6, 11, 22) (OTU (42, 81, (31, 47), 55, 61) from *Acidobacteria* (Table S2). In the group with N addition, keystone taxa included *Anaerolineaceae* (OTU24, 3193, 89, 164, 510, 8, 135, 93) from *Chloroflexi*, *Betaproteobacteriales* (OTU110, 69), *Methylococcales* (OTU71) from *Gammaproteobacteria*, *Myxococcales* (OTU43,112), *Desulfobacterales* (OTU122), and *Desulfuromonadales* (OTU64) from *Deltaproteobacteria*, *Sphingobacteriales* (OTU29) and *Chitinophagales* (OTU80) from *Bacteroidetes*, *Nitrospirae_4-29-1* (OTU6) and *Chthoniobacterales* from *Verrucomicrobi* (Table S2).

### Relationship between soil bacterial community and soil physicochemical properties

**Fig. 5:**
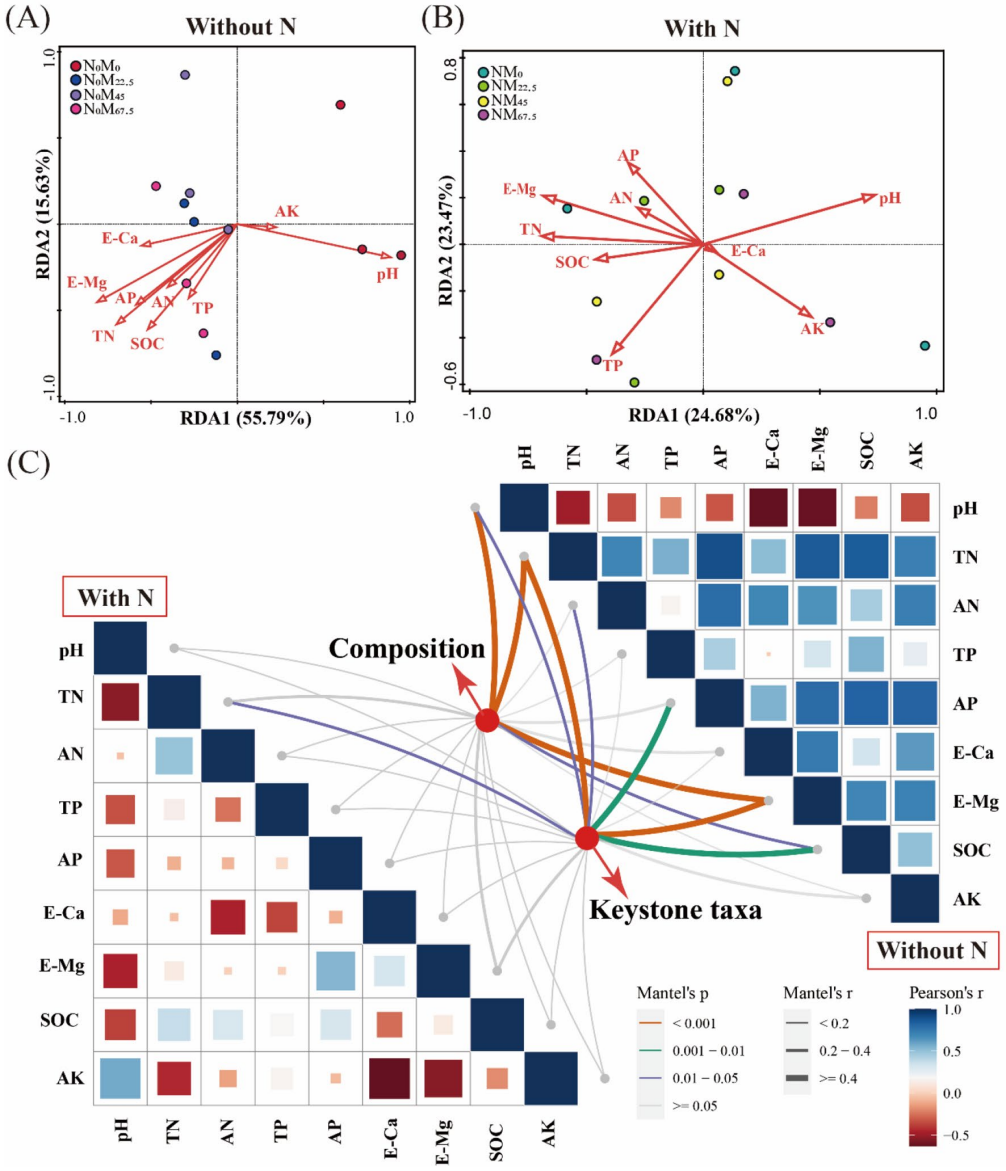
(**A**-**B**) RDA analysis of the correlation between bacterial composition and environmental factors. (**C**) Mantel test analysis showing the correlation between bacterial composition, keystone taxa, and environmental factors

The RDA analysis showed that environmental variables explained 71.42% and 50.15% of variations in bacterial communities in the group without N addition and the group with N addition, respectively (Fig. 5A, B). The pH (F = 8.2, *p* = 0.006), E-Mg (F = 6.8, *p* = 0.004), TN (F = 4.9, *p* = 0.014), AP (F = 3.1, *p* = 0.022), and SOC (F = 2.8, *p* = 0.046) significantly effected soil bacterial community structure in the group without N addition (Fig. 5A). Only pH (F = 2.1, *p* = 0.006) and E-Mg (F = 2.0, *p* = 0.038) significantly affected soil bacterial community structure in the group with N addition (Fig. 5B).

Mantel test analysis suggested that soil bacterial community composition was significantly affected by soil environmental factors, including soil pH, TN, SOC, and E-Mg (*p* < 0.05), while there was no significant effect of environmental factors on soil bacterial community after the addition of N fertilizer (Fig. 5C). Furthermore, in the group without N addition, keystone taxa were positively correlated with soil environmental factors such as pH, TN, AN, AP, SOC and E-Mg. (*p* < 0.05). In the group with N addition, keystone taxa were only positively correlated with soil TN (*p* < 0.05).

### Relationship between rice yield and abiotic and biological factors

**Fig. 6:**
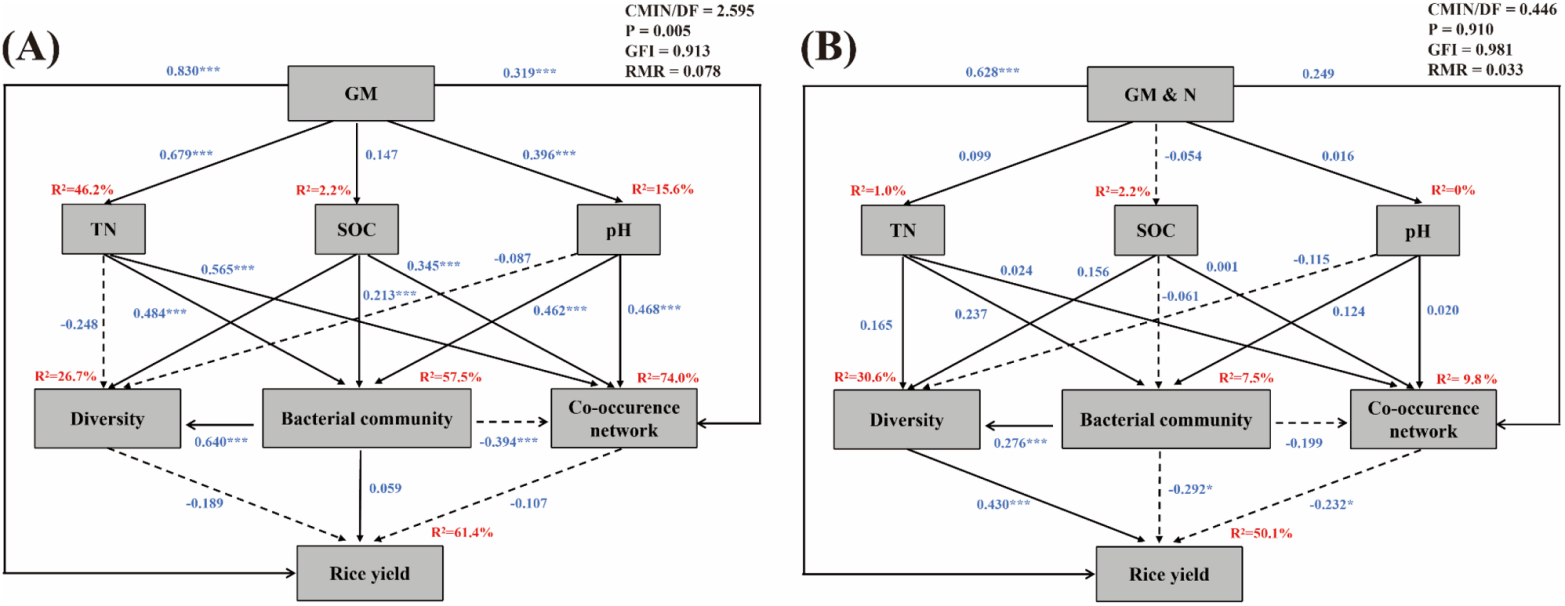
Structural equation modeling (SEM) describing the biotic and abiotic factors affecting rice yields. (**A**-**B**) The SEM of the group without N addition (N_0_M_0_, N_0_M_22.5_, N_0_M_45_, and N_0_M_67.5_) and the group with N addition (NM_0_, NM_22.5_, NM_45_, and NM_67.5_). The direct and indirect effects of different fertilization regimes, soil TN, SOC, pH, bacterial community, and the co-occurrence network on rice yields. Solid lines: positive correlations; dashed line: negative correlations. Numbers above or below the arrow lines are indicative of the correlations. χ^2^, Chi-square; df, degrees of freedom; P, probability level; GFI, RMR are the goodness-of-fit statistics for each model. Significance levels of each predictor are *P < 0.05, **P < 0.01, ***P < 0.001

The SEM analysis further confirmed that fertilization regimes had a direct impact on rice yield (Fig. 6). In the group without N addition, the application of green manure alone significantly altered soil TN and pH, subsequently influencing the overall bacterial community and their interaction. This suggests that fertilization affected the soil environment, subsequently shaping the microbial community. However, the influence of the microbial community on rice yield was not statistically significant (Fig. 6A). Conversely, in the group with N addition, fertilization did not significantly affect soil TN, SOC, and pH. Still, the overall microbial community, diversity, and their interaction significantly impacted rice yield, displaying an opposing effect compared to green manure application alone. These findings indicated that the application of green manure combined with N fertilizer altered the microbial community and rice yield. (Fig. 6B).

## Discussions

The prolonged use of chemical fertilizers can detrimentally affect the soil environment and delicate agricultural ecosystems in karst regions [43–45]. To mitigate these harmful effects and increase rice yield, reducing the input of chemical fertilizers and exploring alternative options such as green manure are necessary. Green manure has been proven effective in maintaining soil health and increasing rice yield [3]. In this study, we observed a significant increase in rice yield ranging from 15.51 to 22.08% with green manure alone, compared to N_0_M_0_, and further improvement of 21.84 to 35% following N fertilizer addition (Fig. 1). This finding is consistent with the previous studies [46–48]. The enhanced rice yield may be attributed to two main factors: (i) improvement in soil fertility and nutrient availability facilitated by the decomposition of green manure [2, 3, 7]; and (ii) alterations in the soil microbial community structure, especially specific functional microbial communities that improve soil microenvironment and nutrient availability, consequently boosting rice yield [15–17].

### Effects of different amounts of green manure on soil physicochemical properties

Research has shown that incorporating green manure into paddy field enhanced rice growth and improved its nutrient absorption and utilization efficiency, ultimately increasing rice yield [49]. In our study, escalating green manure input increased soil TN, AN, AP, and AK contents in the group without N addition, a trend not observed in the group with N addition (Table 1). This rise can be attributed to N release from the decomposition of green manure, which included fixed atmospheric and vegetal N, thereby significantly augmenting N input into the soil [50]. However, the TN and AN showed a decrease with escalating green manure input, consistent with previous findings indicating that excessive N application reduced soil TN and AN contents, suggesting that NM_22.5_ can mitigate N loss [4].

High levels of Ca^2+^ combined with H_2_PO_4_^-^ to form insoluble phosphate in karst soil, limiting phosphorus available for plant uptake [51]. However, the content of AP increased with green manure input, and this effect was further amplified by N addition (Table 1). The application of green manure, especially when combined with N fertilizer, significantly enhanced the abundance and activity of phosphate-solubilizing bacteria. This facilitated the conversion of inorganic phosphorus into organic forms in the soil, thereby substantially increasing available phosphorus content [52]. Additionally, the rhizosphere of green manure released substantial organic acids, interacting with ligand groups on mineral surfaces, thereby enhancing soil potassium availability [53]. Yu et al. reported that an appropriate N application rate promoted phosphorus and potassium uptake in rice, wheres excessive N application diminished this ability [7]. Soil AK and AP were notably reduced in NM_22.5_ and NM_45_ treatments, indicating that a lower amount of green manure combined with N fertilizer enhances the efficient utilization of AK and AP by rice.

We observed no significant change in SOC (*p* > 0.05), indicating that green manure retained stable carbon levels in the soil. This stability could be attributed to the addition of exogenous organic matter and N fertilizer, which stimulated soil microorganisms to mineralize SOC, with recalcitrant components stably stored within soil aggregates in the paddy field [54, 55]. Moreover, the decomposition of green manure and released of N fertilizer resulted in a reduction in soil pH. This acidification process can counterbalance the alkaline nature of lime soil, directly or indirectly impacting the activities and diversity of microbial communities [56].

### Effects of different amounts of green manure on soil bacterial community structure, co-occurrence network and keystone taxa

The distribution pattern of soil bacteria varies under different fertilization regimes due to differences in physicochemical properties [57, 58]. Compared to the application of green manure alone, soil bacterial diversity decreased after the addition of N fertilizer (Fig. 3A), consistent with numerous previous studies linking this decrease to soil pH reduction, which strongly influences microbial community and diversity [19, 31, 59]. Microbial growth is generally constrained by resource availability [60], meaning that the ability of soil microorganisms to access nutrients depends partly on the choice of fertilization methods. An extreme imbalance between available resources and microbial nutrient requirements can lead to changes in soil bacterial communities and their survival strategies [61]. In this study, we observed that the relative abundance of *Chloroflexi*, *Planctomycetes* and *Verrucomicrobia* increased in the group without N addition (Fig. 2A). These bacteria are classified as oligotrophs, capable of thriving under low substrate concentrations [62, 63], suggesting that green manure decomposition provided low-quality nutrients for paddy fields. Conversely, the addition of exogenous inorganic N rapidly alters the soil nutrient status, resulting in a lower C/N ratio compared to green manure alone [15, 18]. *Proteobacteria* and *Bacteroidetes* are considered potential copiotrophs [64], increased in the group with N addition (Fig. 2A), indicating their competitive advantages in nutrient-rich environments [65]. Therefore, we conclude that differences in fertilization affect soil bacterial community composition and their survival strategies.

PCA analysis revealed significant differences in soil bacterial communities under the application of green manure alone. However, a considerable overlap was observed after the addition of N fertilizer (Fig. 3B-D), indicating that N fertilizer disrupted the stability of the original bacterial community compared to the application of green manure alone, leading to homogenization of the soil bacterial community. Soil pH exhibited a strong correlation with soil bacterial community in all treatment (Fig. 5A, B), largely limiting the niche range for the soil bacterial community [66]. The SOC, TN, AN, and AP were significantly correlated with soil bacterial community in the group without N addition (Fig. 5A). Although the nutrients released by green manure decay provided the necessary C, N, P, and K sources for soil microorganisms and rice, the available nutrients were preferentially absorbed and utilized by rice [7], making these environmental factors key constraints on the changes in soil bacterial communities. Microorganisms respond quickly to drastic environmental changes, adjusting their communities and ecological functions to maintain ecosystem stability [67]. In this study, the addition of N fertilizer caused significant disturbance to the soil environment and resulted in the aggregation of soil bacterial community (Fig. 3D). Moreover, a large amount of N input disrupted the original nutrient distribution pattern and caused a priming effect, leading to a lower C/N ratio in the soil, resulting in changes in life history strategies [68–70]. Furthermore, the N addition could enhance the function of microbial taxa that specifically decompose organic compounds, facilitating material transformation and nutrient availability [71, 72]. Therefore, further exploration of the effects of key functional species on nutrient turnover under different fertilization treatments is necessary.

### Effects of different amounts of green manure on soil bacterial co-occurrence network and keystone taxa

Fertilization practices have been shown to alter the stability of microbial networks and the function of keystone taxa [15, 17], which are directly related to nutrient cycling in paddy ecosystems [73]. In this study, we found that the addition of N fertilizer reduced the positive correlation ratio and complexity of soil bacterial network compared with the application of green manure alone (Fig. 4). This suggested a synergistic effect of soil bacteria under the action of green manure alone, while a competitive effect occurred with the addition of N fertilizer [42]. Yuan et al. [74] reported that network complexity and stability strongly influence microbial community structure and ecosystem functional process. The observed decrease in complexity and stability of the bacterial community may lead to changes in keystone taxa and their function within the network patterns under different fertilization regimes.

*Anaerolineales*, *Myxococcales*, *Desulfobacterales*, *Pirellulales*, and *Betaproteobacteriales* are commonly found in the anaerobic environment of rice paddy field [75]. Specially, *Rhizobiales* coexist with leguminous plants and facilitate soil fertility through N fixation [22] *Latescibacteria* play a significant role in decomposing green manure due to their strong saprophytic features, enabling them to degrade polysaccharides, lipids, and proteins in bacterial, plant, and fungal materials [76]. The subgroup (4,5,6,11,22) of *Acidobacteria*, classified as oligotrophs, efficiently utilize limited nitrogen and unstable carbon sources, benefiting from the application of green manure for R strategy propagation [64], and exhibit a high growth rate in response to environmental disturbances [77]. The N-fixed bacteria coexisting with leguminous green manure and other keystone taxa appeared to be important in carbon compound degradation, providing a continuous N supply from atmospheric N and the decomposition of green manure for rice.

In contrast, *Sphingobacteriales* and *Chitinophagalesy*, classified as copiotrophs, thrive on unstable carbon and abundant nitrogen sources [64]. *Nitrospirae_4–29* and *Chthoniobacterales* of *Verrucomicrobia* appear to be important in nitrification and denitrification, respectively [78, 79], indicating that keystone species under the combination of green manure and chemical fertilizer may impact the N transformation process. Mantel’s test analysis showed that a strong correlation between keystone taxa and TN content (Fig. 5C), indicating that the keystone species may positively affect the N use efficiency of rice and significantly increased rice yield [52, 80]. The addition of N fertilizer promoted the aggregation of specific microorganisms and the secretion of hydrolase enzymes, facilitating soil N rotation and enhancing the nitrogen cycling process [26]. Moreover, the combination of green manure with N fertilizer was beneficial in increasing the number of phosphorus-lipolytic bacteria, greatly improving the availability of phosphorus in the soil [52]. Overall, the changes in the microbial network and the function of keystone taxa in different fertilization regimes might have strong effects on nutrient transformation process and, consequently, nutrient use efficiency in rice cultivation.

### Response of biological and abiotic factors on rice yield

The impact of different fertilization regimes on rice yield is substantial, and understanding the microbial mechanisms involved is essential for unraveling the complexity of paddy ecosystems. SEM analysis revealed that the application of green manure significant effected soil TN and pH, consequently shaping soil microbial communities and the co-occurrence network (Fig. 6A). However, this did not result in a significant improvement in rice yield, primarily due to the limited availability of nutrients, which left both soil microorganisms and rice in a state of nutrients starvation [18, 81]. In contrast, when green manure was combined with N fertilizer, there was no significant effect on TN, SOC, and pH, but the soil bacterial community significantly influenced rice yield (Fig. 6B). This varied that N fertilizer improved rice yield by modulating the bacterial community and keystone taxa, which in turn regulated N transformation processes and indirectly promoted nutrient absorption by rice. However, it’s worth noting that while green manure plays a positive role in increasing rice yield, excessive input of green manure provides little additional benefit to rice enhancement. Therefore, the judicious addition of chemical fertilizer on top of green manure incorporation can effectively boost rice yield. These research findings are not only crucial for the sustainability of agricultural production but also deepen our understanding of the relationship between soil microbes and rice growth.

## Conclusion

Compared to the N_0_M_0_ treatment, the application of varying amounts of green manure combined with N fertilizer altered the soil bacterial community and significantly enhanced rice yield in karst paddy areas. The application of green manure alone provided a pristine nutrient source for rice through self-decomposition and symbiosis with nitrogen-fixing bacteria, leading to increases in soil TN, AN, AK, and AP. Conversely, the application of a large amount of N fertilizer reduced the soil C: N ratio, triggering destabilization of the native soil bacterial community. Additionally, keystone taxa shifted from their original roles in N-fixing (*Rhizobiales*) and carbon-degradation (*Latescibacteria* and subgroups of *Acidobacteria*) to functions associated with carbon degradation (*Sphingobacteriales* and *Chitinophagalesy*), nitrification (*Nitrospirae_4–29*), and denitrification (Chthoniobacterales). This alteration in soil community composition and function likely plays a crucial role in enhancing nutrient utilization efficiency in rice, consequently significantly increasing rice yield. The study concludes by advocating for future investigations to focus on specific core taxa to gain a deeper understanding of the roles of soil microorganisms and their metabolic activities in influencing soil properties and rice productivity.

### Electronic supplementary material

Below is the link to the electronic supplementary material.

Supplementary Material 1

## Data Availability

Sequence data that support the findings of this study have been deposited in the https://www.ncbi.nlm.nih.gov/, and the number is PRJNA1031136, SRR26535658 - SRR26535681 (24 records).
