# Supplementary material for: The application of varying amount of green manure combined with nitrogen fertilizer altered the soil bacterial community and rice yield in karst paddy areas
Source: BMC Plant Biol. 2024 Jul 8;24:646. doi: 10.1186/s12870-024-05351-7 (PMC11229212; doi:10.1186/s12870-024-05351-7)
Supplement: Supplementary file 1 — Supplementary Material 1 [file 12870_2024_5351_MOESM1_ESM.docx]

**Supplementary Information**

**Table S1. The elements of co-occurrence network patterns in different treatments.**

| Treatment | Node | Total edge | PPC | Modularity | Average degree |
| --- | --- | --- | --- | --- | --- |
| N_0_M_0_ | 182 | 5492 | 51.78% | 0.642 | 60.022 |
| N_0_M_22.5_ | 174 | 5279 | 53.31% | 0.575 | 60.678 |
| N_0_M_45_ | 180 | 5381 | 54.19% | 0.591 | 59.789 |
| N_0_M_67.5_ | 177 | 4809 | 55.60% | 0.642 | 54.339 |
| NM_0_ | 167 | 5003 | 53.07% | 0.578 | 59.961 |
| NM_22.5_ | 162 | 4367 | 52.37% | 0.617 | 53.914 |
| NM_45_ | 170 | 4657 | 51.69% | 0.647 | 54.788 |
| NM_67.5_ | 175 | 4979 | 52.36% | 0.631 | 56.903 |
| Without N | 125 | 1113 | 67.30% | 0.429 | 17.808 |
| With N | 130 | 913 | 61.66% | 0.365 | 14.046 |

The PPC means the proportion of positive correlation.

**Table S2** **Relevant elements of keystone taxa.**

| **Without N**  **(OTUs)** | **Abundance** | **Degree** | **Closeness**  **centrality** | **Z-score** | **Phylum** | **Class** | **Order** | **Family** | **Genus** |
| --- | --- | --- | --- | --- | --- | --- | --- | --- | --- |
| OTU_91 | 0.16% | 37 | 0.5210 | 2.2541 | Latescibacteria | **/** | **/** | **/** | **/** |
| OTU_431 | 0.12% | 27 | 0.4662 | 1.0791 | Latescibacteria | **/** | **/** | **/** | **/** |
| OTU_19 | 0.91% | 34 | 0.5103 | 1.9022 | Chloroflexi | Anaerolineae | Anaerolineales | Anaerolineaceae | **/** |
| OTU_20 | 0.60% | 31 | 0.4921 | 1.5495 | Chloroflexi | Anaerolineae | Anaerolineales | Anaerolineaceae | **/** |
| OTU_93 | 0.31% | 27 | 0.4644 | 1.0789 | Chloroflexi | Anaerolineae | Anaerolineales | Anaerolineaceae | Anaerolinea |
| OTU_111 | 0.22% | 34 | 0.5082 | 1.9020 | Proteobacteria | Deltaproteobacteria | Myxococcales | Haliangiaceae | Haliangium |
| OTU_62 | 0.35% | 33 | 0.5167 | 1.7861 | Proteobacteria | Alphaproteobacteria | Rhizobiales | Xanthobacteraceae | Pseudolabrys |
| OTU_125 | 0.12% | 32 | 0.5000 | 1.6673 | Proteobacteria | Gammaproteobacteria | Betaproteobacteriales | Burkholderiaceae | **/** |
| OTU_110 | 0.29% | 29 | 0.4788 | 1.3143 | Proteobacteria | Gammaproteobacteria | Betaproteobacteriales | Rhodocyclaceae | Dechloromonas |
| OTU_63 | 0.46% | 27 | 0.4901 | 1.0819 | Proteobacteria | Gammaproteobacteria | Betaproteobacteriales | Gallionellaceae | Sideroxydans |
| OTU_28 | 0.63% | 31 | 0.5000 | 1.5505 | Proteobacteria | Gammaproteobacteria | Ectothiorhodospirales | Thioalkalispiraceae | Thioalkalispira |
| OTU_70 | 0.27% | 29 | 0.4882 | 1.3154 | Proteobacteria | Deltaproteobacteria | Myxococcales | Archangiaceae | Anaeromyxobacter |
| OTU_101 | 0.12% | 29 | 0.4882 | 1.3154 | Proteobacteria | Deltaproteobacteria | Desulfarculales | Desulfarculaceae | Desulfatiglans |
| OTU_5 | 0.58% | 33 | 0.4960 | 1.7837 | Planctomycetes | Planctomycetacia | Pirellulales | Pirellulaceae | Pirellula |
| OTU_51 | 0.15% | 31 | 0.4940 | 1.5498 | Planctomycetes | Pla4 lineage | **/** | **/** | **/** |
| OTU_102 | 0.14% | 31 | 0.4901 | 1.5493 | Gemmatimonadetes | Gemmatimonadetes | Gemmatimonadales | Gemmatimonadaceae | **/** |
| OTU_31 | 0.71% | 29 | 0.4980 | 1.3165 | Acidobacteria | Subgroup 6 | **/** | **/** | **/** |
| OTU_47 | 0.19% | 29 | 0.4960 | 1.3163 | Acidobacteria | Subgroup 22 | **/** | **/** | **/** |
| OTU_42 | 0.20% | 29 | 0.4509 | 1.3110 | Acidobacteria | Subgroup 4 | **/** | **/** | **/** |
| OTU_55 | 0.14% | 28 | 0.4940 | 1.1992 | Acidobacteria | Subgroup 11 | **/** | **/** | **/** |
| OTU_61 | 0.13% | 27 | 0.4788 | 1.0806 | Acidobacteria | Subgroup 22 | **/** | **/** | **/** |
| OTU_81 | 0.23% | 27 | 0.4788 | 1.0806 | Acidobacteria | Subgroup 5 | **/** | **/** | **/** |
| **With N**  **(OTUs)** | **Abundance** | **Degree** | **Closeness**  **centrality** | **Z-score** | **Phylum** | **Class** | **Order** | **Family** | **Genus** |
| OTU_24 | 1.04% | 34 | 0.5020 | 2.6296 | Chloroflexi | Anaerolineae | Anaerolineales | Anaerolineaceae | **/** |
| OTU_3193 | 0.30% | 24 | 0.4468 | 1.3106 | Chloroflexi | Anaerolineae | Anaerolineales | Anaerolineaceae | **/** |
| OTU_89 | 0.15% | 26 | 0.4649 | 1.5753 | Chloroflexi | Anaerolineae | Anaerolineales | Anaerolineaceae | **/** |
| OTU_164 | 0.12% | 23 | 0.4615 | 1.1814 | Chloroflexi | Anaerolineae | Anaerolineales | Anaerolineaceae | **/** |
| OTU_510 | 0.18% | 26 | 0.4684 | 1.5758 | Chloroflexi | Anaerolineae | Anaerolineales | Anaerolineaceae | **/** |
| OTU_8 | 1.12% | 30 | 0.4884 | 2.1031 | Chloroflexi | Anaerolineae | Anaerolineales | Anaerolineaceae | **/** |
| OTU_135 | 0.17% | 27 | 0.4809 | 1.7086 | Chloroflexi | Anaerolineae | Anaerolineales | Anaerolineaceae | Anaerolinea |
| OTU_93 | 0.35% | 24 | 0.4532 | 1.3115 | Chloroflexi | Anaerolineae | Anaerolineales | Anaerolineaceae | Anaerolinea |
| OTU_5 | 0.83% | 33 | 0.4980 | 2.4979 | Planctomycetes | Planctomycetacia | Pirellulales | Pirellulaceae | Pirellula |
| OTU_110 | 0.33% | 32 | 0.4941 | 2.3662 | Proteobacteria | Gammaproteobacteria | Betaproteobacteriales | Rhodocyclaceae | Dechloromonas |
| OTU_69 | 0.29% | 30 | 0.4922 | 2.1036 | Proteobacteria | Gammaproteobacteria | Betaproteobacteriales | Burkholderiaceae | **/** |
| OTU_71 | 0.23% | 30 | 0.4903 | 2.1034 | Proteobacteria | Gammaproteobacteria | Methylococcales | Methylomonaceae | Methylomicrobium |
| OTU_43 | 0.18% | 30 | 0.4865 | 2.1029 | Proteobacteria | Deltaproteobacteria | Myxococcales | bacteriap25 | **/** |
| OTU_112 | 0.18% | 25 | 0.4516 | 1.4424 | Proteobacteria | Deltaproteobacteria | Myxococcales | Haliangiaceae | Haliangium |
| OTU_122 | 0.12% | 28 | 0.4737 | 1.8389 | Proteobacteria | Deltaproteobacteria | Desulfobacterales | Desulfobacteraceae | Sva0081 sediment group |
| OTU_64 | 0.90% | 23 | 0.4406 | 1.1786 | Proteobacteria | Deltaproteobacteria | Desulfuromonadales | Geobacteraceae | Geobacter |
| OTU_29 | 0.33% | 27 | 0.4755 | 1.7079 | Bacteroidetes | Bacteroidia | Sphingobacteriales | Lentimicrobiaceae | **/** |
| OTU_80 | 0.28% | 27 | 0.4755 | 1.7079 | Bacteroidetes | Bacteroidia | Chitinophagales | Chitinophagaceae | **/** |
| OTU_6 | 1.28% | 23 | 0.4565 | 1.1807 | Nitrospirae | 4-29-1, | **/** | **/** | **/** |
| OTU_195 | 0.14% | 22 | 0.4565 | 1.0495 | Verrucomicrobia | Verrucomicrobiae | Chthoniobacterales | Chthoniobacteraceae | Candidatus Udaeobacter |


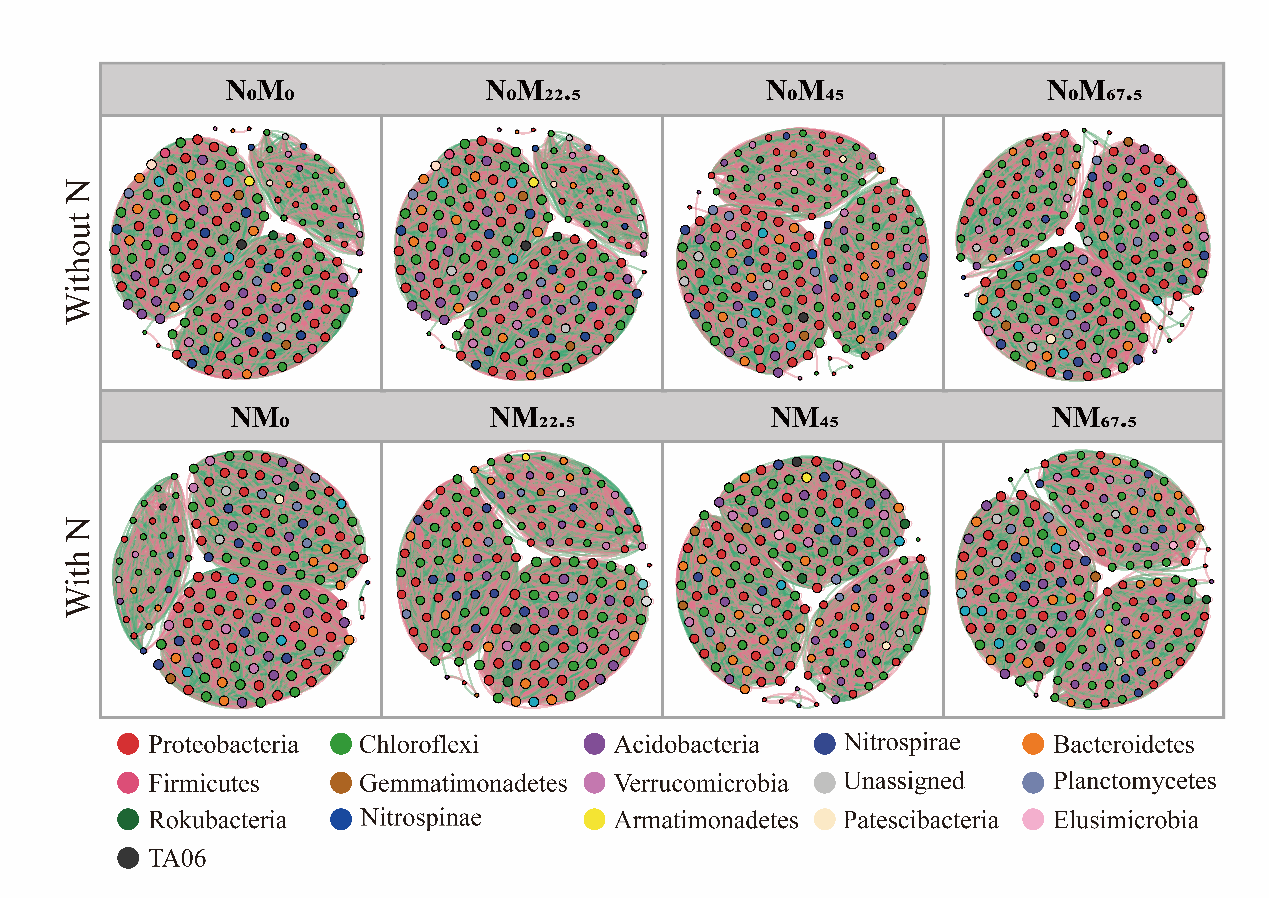


**Fig. S1 Network visualizes the interactions between soil operational taxonomic units (OTUs) in different fertilization treatments. The nodes are colored according to different species categories. Positive correlations are displayed in red, whereas negative correlations are displayed in green.**
